# Pretreatment neutrophil to lymphocyte ratio as prognostic factor in metastatic breast cancer treated with cyclin dependent kinase 4/6 inhibitors

**DOI:** 10.3389/fonc.2022.1105587

**Published:** 2023-01-19

**Authors:** Pauline Rottier, George Emile, Alison Johnson, Christelle Levy, Djelila Allouache, Ioana Hrab, Carine Segura, Adeline Morel, Maud Villemin, Coraline Dubot-Poitelon, Louis Boismoreau, François Cherifi, Justine Lequesne, Angélique Da Silva

**Affiliations:** ^1^ Breast Cancer Unit, François Baclesse, Comprehensive Cancer Center Institut Normand du Sein, Caen, France; ^2^ Department of Clinical Research, Francois Baclesse Comprehensive Cancer Center, Caen, France

**Keywords:** metastatic breast cancer, NLR, cyclin dependent kinase inhibitor, prognostic factor, hormone dependent cancer

## Abstract

**Background:**

Cyclin dependent kinase inhibitors (CdK4/6i) changed the course of hormone receptor positive (HR+) HER2 negative (HER2-) metastatic breast cancer (mBC). To date, no factors have been shown to predict response to CdK4/6i. Neutrophil-to-lymphocyte ratio (NLR), an indicator of the host systemic inflammatory response, is an independent prognostic factor for survival in cancers. We conducted this study to evaluate the impact of NLR on survival in mBC patients treated with first line CdK4/6i.

**Methods:**

All mBC patients treated with first line CdK4/6i between November 2015 and December 2019 were retrospectively included. The biomarker threshold was defined using ROC curves. We analyzed progression free survival (PFS), overall survival (OS), 12-month PFS and response rate according to NLR in univariable and multivariable analysis.

**Results:**

A total of 126 patients treated with palbociclib (n=101), ribociclib (n=18) or abemaciclib (n=7) were included, with a median follow-up of 33 months [range: 2.9–57]. Median age was 65 years [29-86], 40% patients had good performance status (ECOG-PS 0). Most patients (71%) were included at the metastatic relapse stage and 29% had only bone metastases. Median PFS and median OS were 27 and 51 months, respectively. High NLR (≥ 2.53) was significantly associated with worse PFS (Hazard Ratio (HR)=0.50, CI_95%_ = [0.32–0.79]) and worse OS (HR=0.45, [CI_95%_: 0.23–0.87]). In multivariable analysis, NLR and ECOG PS were independently factors associated with PFS (p=0.016 and p=0.001, respectively).

**Conclusion:**

High NLR was associated with worse PFS and OS in HR+ HER2- mBC patients treated with first line CdK4/6i. NLR is a reliable and inexpensive prognostic marker, easily accessible in routine clinical practice, which could help optimize the therapeutic strategy. These results need to be confirmed in larger prospective studies.

## Introduction

Breast cancer (BC) is the most common malignancy among women and one of the leading causes of death by cancer worldwide (1) despite effective early detection methods and new therapeutic advances. Around 6-10% of BC are diagnosed with *de novo* metastatic disease and 25-30% present a metastatic relapse (2). Metastatic breast cancer (mBC) has a poor survival with a 5-year relative survival rate dropping to around 38% vs. 96% for early BC (eBC), in Europe (3). Approximately 70% of BC are hormone receptor positive (HR+) and human epidermal growth factor receptor 2 negative (HER2-). Endocrine therapy (ET) is the main treatment for patients with HR+/HER2- mBC. The advent of cyclin-dependent kinase inhibitors (CdK4/6i) has considerably improved the prognosis. They are now the gold standard for first line treatment of HR+/HER2- mBC without extensive visceral involvement (3–6).

Prognostic factors are important in estimating outcomes and identifying the optimal treatment for each patient. Some clinical or histological markers are commonly used and validated in HR+/HER2- mBC such as poor Eastern Cooperative Oncology Group Performance Status (ECOG-PS), higher tumor grade and Ki67 expression, negative progesterone receptor (PR) status, prior therapy, sites and number of metastases (multiple vs single), and shorter time to progression to mBC (7). The choice of first-line treatment is crucial, as it affects patients’ outcome. However, until now no predictive factor of response to CdK4/6i and ET has been identified. Novel biomarkers are needed to help personalize first line treatment.

Over the last decade, host systemic inflammatory response have been shown to be involved in tumor growth, invasion, angiogenesis and progression (8, 9). This inflammation could be assessed by pretreatment peripheral differential leukocyte count with estimation of lymphocyte count and the calculation of more informative ratios such as neutrophil-to-lymphocyte ratio (NLR), platelet-to-lymphocyte ratio (PLR), lymphocyte-to-monocyte ratio (LMR) and estimation of lymphocyte count. Several studies in different stages of solid cancers (10–12), including BC, evaluated these ratios and they are now acknowledged as predictive and prognostic factors. In a metanalysis, it was highlighted that high pretreatment NLR was an independent poor prognostic factor for overall survival (OS) and progression-free survival (PFS) in all-stage BC, with the strongest association in the HR+/HER2- subgroup (13). Koh et al. (14) revealed in a prospective study that both NLR and PLR are independently associated with an increased risk of mortality in all-stage BC. However, these inflammation biomarkers have mostly been evaluated in the (neo)adjuvant chemotherapy setting for eBC (15, 16), particularly in triple-negative BC (TNBC) (17). Data remains limited and inconsistent for mBC (18). High LMR before neoadjuvant chemotherapy was reported as a favorable prognostic factor in eBC regardless of HR/HER2 status (19), but no data has been reported for mBC HR+/HER2-.

The aim of our study was to assess the prognostic impact of NLR, lymphopenia, PLR and LMR on survival and response rates in women receiving first line CdK4/6i in association with ET for locally advanced or mBC.

## Methods

### Population

We carried out a retrospective single center study at the Comprehensive Cancer Center François Baclesse in Caen, France, as recommended by REMARK (REporting recommendations for tumor MARKer prognostic studies) for the evaluation of prognostic tumor marker (20). All adult women who received CdK4/6i for histologically proven HR+/HER2- locally advanced or mBC from November 2015 to December 2019 were included. Patients receiving any of the European Medicines Agency (EMA) or Food and Drug Administration (FDA)-approved CdK4/6i (palbociclib, ribociclib, abemaciclib) in association with ET as first-line treatment were included. Patients were excluded if they had received other prior first-line treatment or presented with visceral crisis.

### Endpoint

We collected the general characteristics of patients (e.g., age, ECOG-PS, menopausal status), their disease (e.g., TNM staging, hormone receptor expression and SBR (Scarff Bloom Richardson) grade from the primary tumor site or a current metastatic lesion) and prior therapy (adjuvant treatment, palliative radiotherapy or corticosteroid therapy). Results of the blood test performed at the latest the week before starting treatment were collected. NLR was defined as the absolute neutrophil count divided by the absolute lymphocyte count, PLR was defined as the absolute platelet count divided by the absolute lymphocyte count and LMR was defined as the absolute lymphocyte count divided by the absolute monocyte count. Lymphopenia was defined by absolute lymphocyte count (ALC) below 1.5 G/L. Tumor imaging (by computed tomography scan) was performed every 3 cycles and disease response was classified by the radiologist according to the Response Evaluation Criteria in Solid Tumors [RECIST, version 1.1 (21)] as complete response (CR), partial response (PR), stable disease (SD) or progressive disease (PD). Objective response rate (ORR) corresponded to the proportion of patients in whom a CR or PR was observed. Disease control rate (DCR) represented the percentage of patients with either CR, PR, or SD as the best overall response. PFS was defined as the time elapsed between CdK4/6i initiation and radiological progression, death or lost to follow-up. Overall survival (OS) was defined as the time elapsed between CdK4/6i initiation and death from any cause. Adverse events (AE) collected at each medical visit were graded according to National Cancer Institute Common Terminology Criteria for Adverse Events (NCI CTCAE) version 5.0.

### Objectives

The primary objective was to assess the PFS according to pretreatment NLR.

Secondary objectives included assessment of 12-month PFS, OS, ORR and DCR according to pretreatment NLR; assessment of PFS and OS according to lymphopenia, PLR and LMR and evaluation of safety.

### Statistical analysis

Descriptive analysis of data provided frequencies and percentages for qualitative variables, and median and extreme values for quantitative variables. Survival curves were estimated by the Kaplan Meier method, and compared by the log-rank test. Multivariable analysis for PFS and OS was performed using Cox’s proportional hazards regression model including biological markers significantly associated with survival at a significance level of 0.10 and adjusted on clinical parameters. A stepwise model selection was performed through Akaike’s Information Criterion optimization, corresponding to significance-based selection at a significance level of 0.157. The optimal cut-off values for the NLR, PLR and LMR to predict 1-year progression were determined by maximizing the product of sensitivity and specificity, through receiver operating characteristics (ROC) curve analysis. The characteristics of high NLR and low NLR patients were compared by χ2 test (or Fisher’s exact test, in case of observed values per category < 5) for the qualitative variables, and by the Student’s t-test for the quantitative variables (or Wilcoxon non-parametric test if data were not normally distributed). Statistical tests and confidence intervals were calculated with an overall risk of 5%. All incident cases were assessed (no calculation of the number of subjects needed). Analyses were conducted using R software, version 4.0.2 (https://cran.r-project.org/bin/windows/base/).

### Ethic

The study was in accordance with national regulations regarding research involving human subjects. Registration in the CIL (Correspondant Informatique et Libertés) register was carried out for this study. Patients non-opposition to the use of their data was sought after verification of vital status. All data were anonymized for statistical analysis.

## Results

### Population

**Figure 1:**
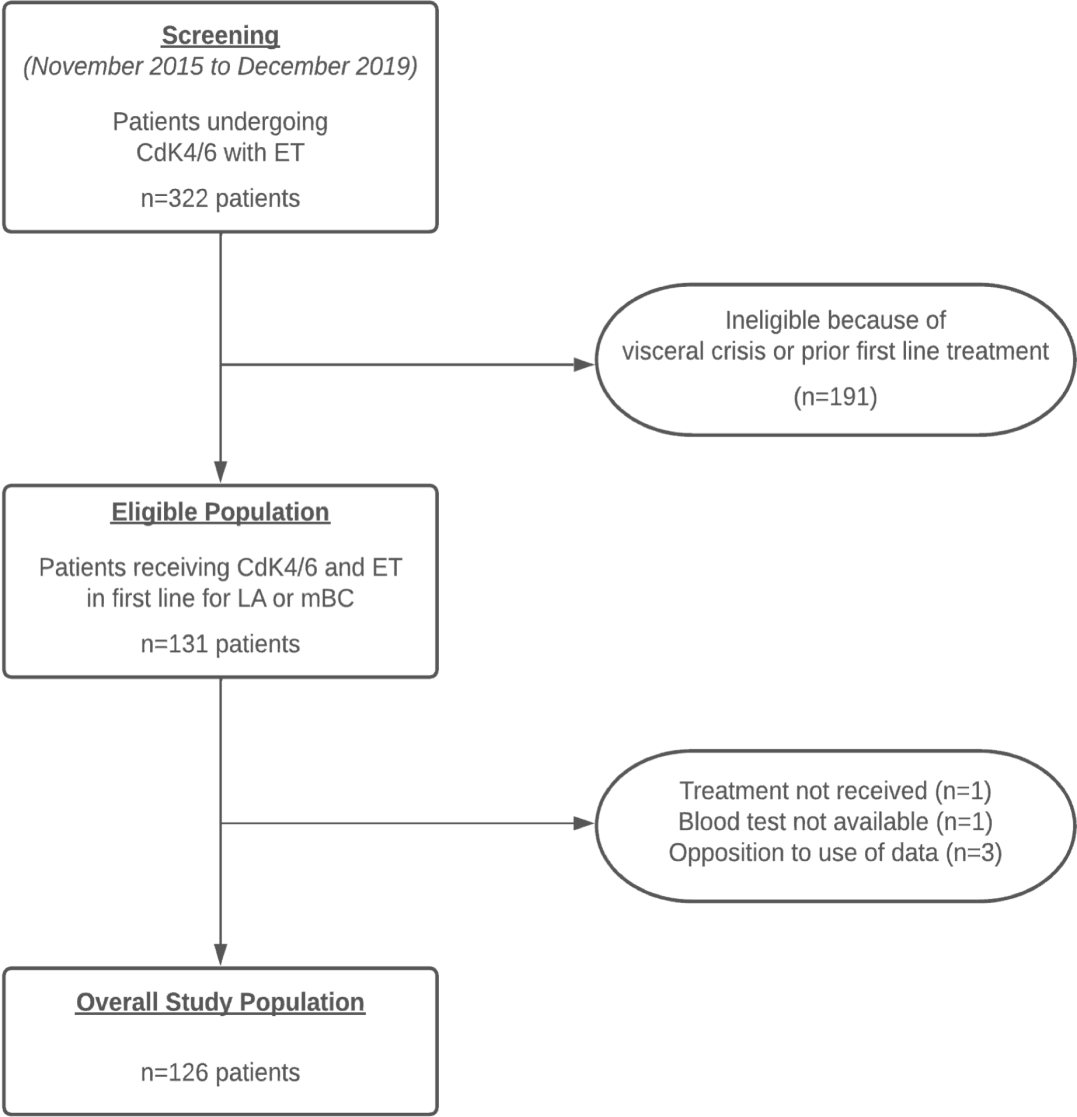
Flow-chart.

From November 2015 to December 2019, 126 patients were included, with a median follow-up of 33 months (range, 2.9 to 57) (**Figure 1**: Flow-chart). The median age at inclusion was 65 years (range, 29 to 86). Thirty-six (28.6%) patients presented with *de novo* mBC. 37 patients (29.4%) had bone metastases only, of whom 14 were *de novo* metastatic. The mean disease-free interval (DFI), time between the end of adjuvant treatment before starting any ET and tumor recurrence, was 124.6 months (range, 1 to 360 months). Only 2 patients, one in each NLR group, received chemotherapy in the year preceding the introduction of treatment (last injection 48 days and 51 days before). Thirty patients (23.8%) received radiotherapy within 90 days of beginning ET and CdK4/6i, for a median time interval of 21 days (range, 3 to 66 days). Ten patients had a concomitant prescription of corticosteroid therapy at the first intake of CdK4/6i, with a mean dosage of 35.5 mg. The most commonly prescribed CdK4/6i was palbociclib (n=101, 80%), followed by ribociclib (n=18, 14%) and abemaciclib (n=7, 6%), combined with ET (aromatase inhibitor +/- LHRH analogue for 104 patients (82.5%) or fulvestrant for 22 patients (17.5%)). To the pretreatment stage, median and range of neutrophil, lymphocyte, platelet, and monocyte counts were 3.46 G/L [1.19;14.73], 1.44 G/L [0.14;4.40], 267 G/L [101;622] and 0.49 G/L [0.10;1.30], respectively. Patient characteristics are presented in **Table 1**.

**Table 1:** Patient characteristics at baseline.

Population characteristics	N = 126 (%)
Median age, years [range]	65 [29;86]
ECOG PS	
0	50 (39.7)
1	62 (49.2)
2	12 (9.5)
3	2 (1.6)
Histology at diagnosis	
Ductal	94 (74.6)
Lobular	29 (23.0)
Other	3 (2.4)
SBR grade at diagnosis	
I	18 (14.3)
II	72 (57.1)
III	32 (25.4)
Unknown	4 (3.0)
Stage at diagnosis	
I	13 (10.8)
II	37 (30.8)
III	34 (27)
IV	36 (28.6)
Unknown	6 (4.8)
Menopause	
Yes	94 (74.6)
No	32 (25.4)
*De novo* metastatic cancer	
Yes	36 (28.6)
No	90 (71.4)
Metastatic sites	
Locoregional only	3 (2.4)
Bone only	37 (29.4)
Others	86 (68.3)
Adjuvant treatment *^a^*	
Yes	87 (69)
No	39 (31)
Radiotherapy within 90 days	
Yes	30 (23.8)
No	96 (76.2)
Corticosteroid therapy *^b^*	
Yes	10 (7.9)
No	116 (92.1)
Blood count (G/L; [range])	
Neutrophils count	3.46 [1.19;14.73]
Lymphocytes count	1.44 [0.14;4.40]
Platelets count	267 [101;622]
Monocytes count	0.49 [0.10;1.30]
NLR (cut-off = 2.53)	
High	64 (51)
Low	62 (49)
Lymphopenia (< 1.5G/L)	
Yes	67 (53)
No	59 (47)
PLR (cut-off = 174.4)	
High	68 (54)
Low	58 (46)
LMR (cut-off = 3.3)	
High	60 (48)
Low	66 (52)

ECOG-PS, Eastern Cooperative Oncology Group – Performance Status; SBR, Scarff-Bloom-Richardson; NLR, Neutrophil to Lymphocyte Ratio; PLR, Platelet to Lymphocyte Ratio; LMR, Lymphocyte to Monocyte Ratio.

a including chemiotherapy and/or radiotherapy and/or endocrine therapy.

b prior or at baseline.

### Overall population outcomes

The median PFS time was 27 months (CI_95%_= [21–36]), with a 12-month PFS rate of 73.8% (CI_95%_= [65.7–81.2]). At the end of the follow-up, 61.9% patients (n=78) progressed with first-line metastatic therapy and 31.7% patients (n=40) died. The median OS was 51 months. DCR was 92.1% (16 RC, 64 PR and 36 SD, i.e. 116 patients) and ORR was 63.5% (80 patients).

### Prognostic value of NLR

**Figure 2:**
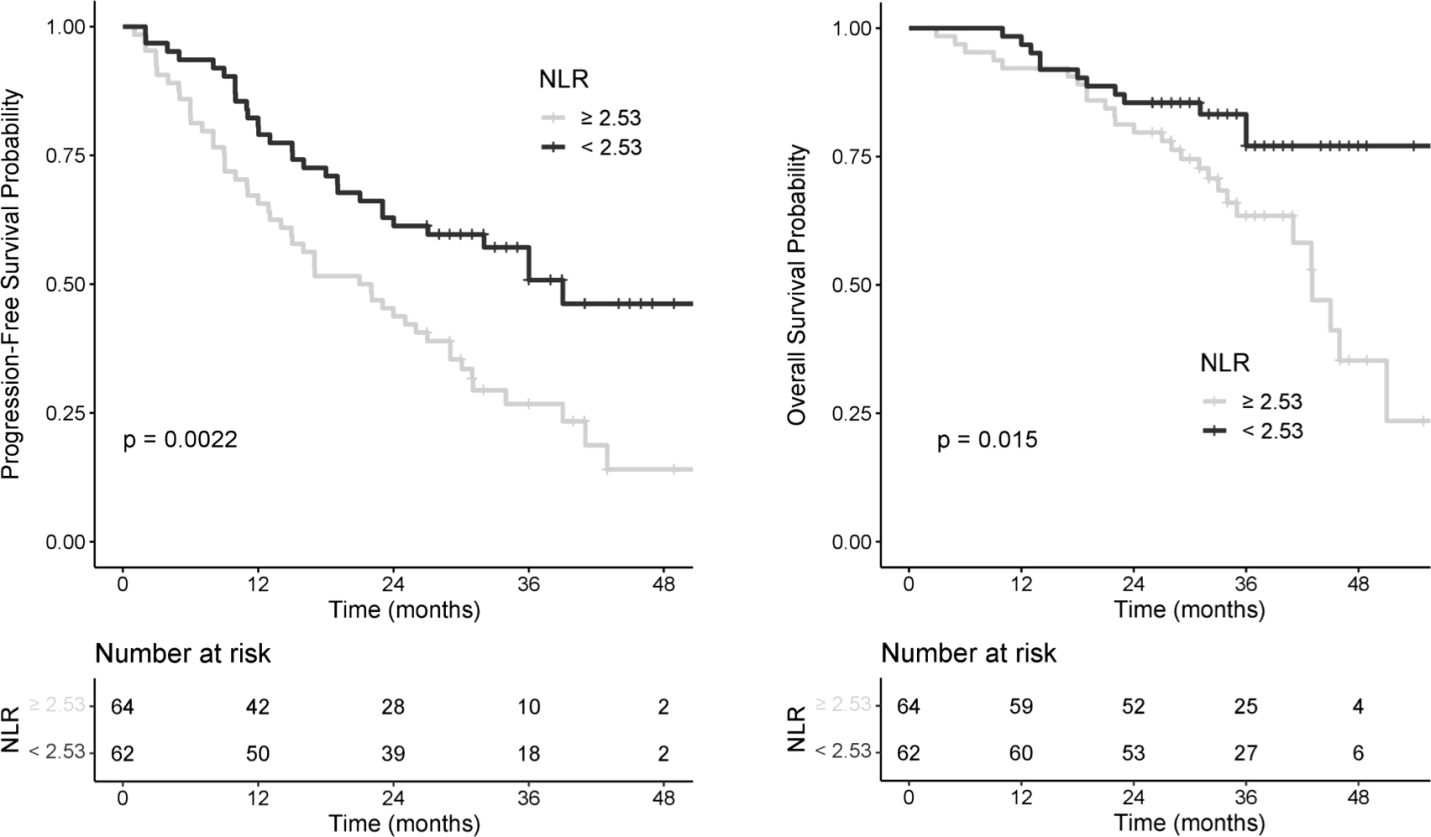
PFS and OS probability according to pretreatment NLR.

The optimal NLR cut-off value to predict progression within 12 months after metastatic diagnosis was 2.53; 64 patients (50.8%) were classified in the high NLR group (NLR ≥ 2.53). The two groups were similar except for pretreatment ECOG-PS and the occurrence of radiotherapy within 90 days. (**Table 2**). PFS was significantly better in the low NLR group (**Figure 2**: PFS and OS probability according to pretreatment NLR) with a median of 39 months compared to the high NLR group with a median of 21.5 months (HR=0.50, [CI_95%_: 0.32–0.79], log-rank p=0.002). The 12-month PFS rate for the low NLR group was 80.7% [CI_95%_: 71.4–91.1] versus 65.5% [CI_95%_: 55.0–78.4] for the high NLR group (**Table 3**). In a subgroup analysis excluding patients who received radiotherapy within 90 days, we observed the same difference of PFS between the two NLR groups with a HR=0.49 ([CI_95%_: 0.29–0.83]) in favor of low NLR group.

**Table 2:** Patient characteristics at baseline according to NLR groups.

Variable	NLR		p value
	High (≥ 2.53) n = 64 (50.8%)	Low (< 2.53) n = 62 (49.2%)	
Median age, years [range]	65 [29;86]	65.5 [32;83]	0.58
ECOG-PS			0.012 ^a^
0	18 (28.1)	32 (51.6)	
1, 2 or 3	46 (71.9)	30 (48.4)	
Postmenopausal patients	50 (78.1)	44 (71)	0.47
Prior therapy for eBC ^1^	46 (71.9)	41 (66.1)	0.69
*De novo* stage IV disease	15 (25)	21 (33.9)	0.39
Bone metastases only	19 (29.7)	18 (29)	1
Existence of visceral metastases	38 (59.4)	33 (53.2)	0.61
Radiotherapy within 90 days	23 (35.9)	7 (11.3)	0.0004 ^a^
Corticosteroid therapy prior or at treatment initiation	4 (6.3)	6 (9.7)	0.53
iCDK 4/6			0.58
Palbociclib	53 (82.8)	48 (77.4)	
Ribociclib	7 (10.9)	11 (17.8)	
Abemaciclib	4 (6.3)	3 (4.8)	
Endocrinotherapy			0.19
AI	15 (23.4)	7 (11.3)	
Fulvestrant	35 (54.7)	38 (61.3)	
AI + LHRH analog	14 (21.9)	17 (27.4)	

eBC, early Breast Cancer; iCDK 4/6, inhibitor of cyclin dependent kinase 4/6; AI, aromatase inhibitor.

^1^including chemotherapy and/or radiotherapy and/or endocrine therapy.

a significant if p<0.05.

**Table 3:** 12-month PFS rate according to biomarkers.

Variable	N	Number at risk	12-month PFS rate	CI_95%_
NLR				
≥ 2.53	64	42	65.6	[55.0 – 78.4]
< 2.53	62	50	80.7	[71.4 – 91.1]
Lymphopenia				
< 1.5 G/L	67	46	68.7	[58.4 – 80.7]
≥ 1.5 G/L	59	46	78.0	[68.1 – 89.3]
PLR				
≥ 174.4	68	47	69.1	[59.0 – 81.0]
< 174.4	58	45	77.6	[67.6 – 89.1]
LMR				
≥ 3.3	60	43	71.7	[61.1 – 84.0]
< 3.3	66	49	74.2	[64.4 – 85.6]

NLR, Neutrophil to Lymphocyte Ratio; PLR, Platelet to Lymphocyte Ratio; LMR, Lymphocyte to Monocyte Ratio.

Low NLR was significantly associated with better OS, HR=0.45 ([CI_95%_: 0.23–0.87], log-rank p=0.015). Median OS was 43 and 56 months for the high NLR and low NLR group, respectively (**Figure 2**: PFS and OS probability according to pretreatment NLR).

Distribution of response was significantly different between the low NLR and the high NLR groups (p=0.041), with better response in the low NLR (**Table 4**). We observed more CR in the low NLR group (n=12, 19.4%) than in the high NLR group (n=4, 6.2%). ORR was 66.2% in the low NLR group and 60.9% in the high NLR group; DCR was 96.8% and 87.5% respectively.

**Table 4:** Best response according to NLR.

Variable	N	CR (%)	PR (%)	SD (%)	PD (%)	p value
**NLR**						0.041^a^
** ≥ 2.53**	64	4 (6.2)	35 (54.7)	17 (26.6)	8 (12.5)	
** < 2.53**	62	12 (19.4)	29 (46.8)	19 (30.6)	2 (3.2)	

NLR, Neutrophil to Lymphocyte Ratio; CR, Complete response; PR, partial Response; SD, Stable disease; PD, Progressive disease.

a p< 0.05.

### Prognostic value of lymphopenia and other ratios

**Figure 3:**
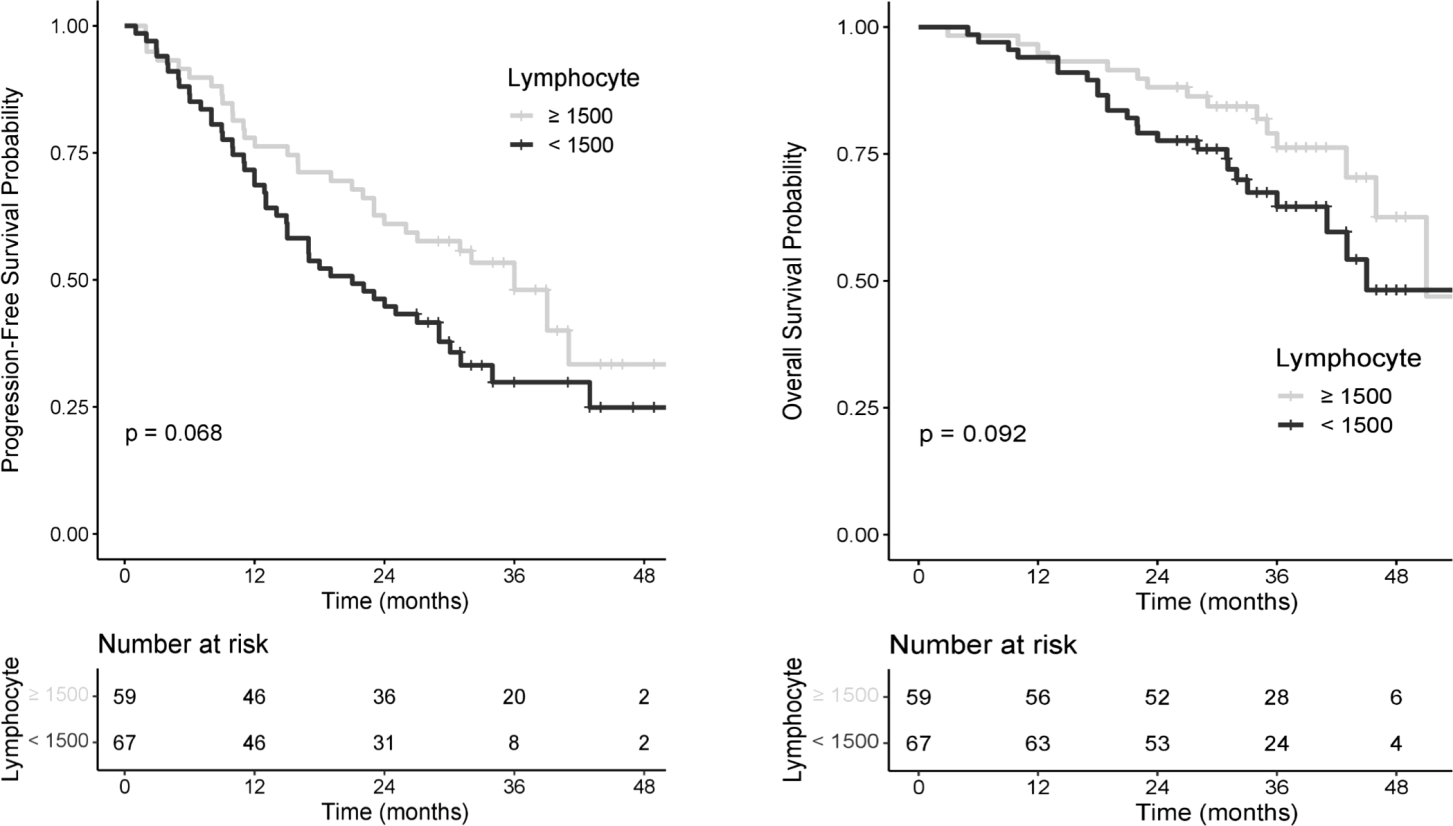
PFS and OS probability according to pretreatment ALC.

Lymphopenia group (n=67 patients) had shorter median PFS, 21 months versus 36 months for patients with normal ALC (HR=0.52, [CI_95%_: 0.30–0.90], log-rank p=0.068) (**Figure 3**: PFS and OS probability according to pretreatment ALC). The 12-month PFS rate was 78.0% [CI_95%_: 68.1–89.3] in the normal ALC group and 68.7% [CI_95%_: 58.4–80.7] in the lymphopenia group (**Table 3**). OS was greater in the normal ALC with a 10 months differential on median OS (51 vs 41 months, HR=0.58 [CI_95%_: 0.30–1.10], log-rank p= 0.09).

The optimal PLR cut-off was 174.4, accounting for 68 patients (54%) in the high PLR group. Pretreatment PLR did not influence PFS (HR=0.73, [CI_95%_: 0.47–1.15], log-rank p=0.17) with median PFS of 22.6 months in the high PLR group and 36 months in the low PLR group (**Table 3**). The optimal LMR cut-off was 3.3, accounting for 60 patients (48%) in the high LMR group. Pretreatment LMR did not influence PFS (HR=0.75, [CI_95%_: 0.48–1.18], log-rank p=0.21), with median PFS of 36 months in the high LMR group and 24.5 months in the low LMR group (**Table 3**). There was no association between PLR or LMR and OS.

### Multivariable analysis

In multivariable analysis, NLR< 2.53 and lymphopenia were included in the model, with adjustment on dose reduction, occurrence of grade 3/4 toxicity, *de novo* metastatic cancer, bone metastases, radiotherapy within 90 days, SBR grade, RP status and ECOG-PS status. Selection model retained NLR< 2.53 and ECOG PS as independently factors associated with PFS, with respectively p=0.016 and p=0.001. NLR< 2.53 was an independent protector factor with a HR=0.57 ([0.36–0.90]). An impaired of general status (ECOG-PS 1, 2 or 3) was associated with worse survival (HR=2.3 [1.37–3.79]) in multivariable Cox model (**Supplementary Table 1**).

### Safety

The most frequently reported AE were hematologic toxicities with neutropenia (n=110 patients, 87.3%), anemia (n=79, 62.7%) and thrombocytopenia (n=38, 30.2%). Grade 3/4 neutropenia was observed in 57 patients (45%). Only few patients experienced grade 3/4 anemia (n=12, 9.5%) and thrombocytopenia (n=2, 1.6%). Dose reductions were required for 52 patients (41.3%). We also reported 7 venous thromboembolism (VTE) events (n=5.6%), including 2 pulmonary embolism (PE) (n=1.6%) and 5 deep vein thrombosis (DVT) (n=4.0%), and all with Palbociclib which represented 6.9% of patients treated with Palbociclib. None stopped treatment. Two patients (n=1.6%) were suspected of developing interstitial lung disease (ILD), both receiving Palbociclib and with high NLR.

## Discussion

Our study highlighted that pretreatment high NLR (≥ 2.53) was a prognostic biomarker associated with worse PFS and OS in women treated with first-line CdK4/6i and ET for metastatic or locally advanced HR+/HER2- breast cancer.

Low NLR appears to be an independent protective factor for PFS and OS with more than 50% risk reduction of progression or death (HR=0.44, [CI_95%_: 0.23–0.87] for OS). Our results are consistent with previous studies. Four recent meta-analyses corroborate our findings showing that NLR is an independent prognostic factor for PFS and OS in patients with BC at different stages (13, 22, 23), especially for luminal A subtype (24). Wariss et al. (25) reported an association between high NLR and worse OS in 2,374 eBC and mBC patients, for patients with luminal subtypes. In another study concerning mTNBC, NLR> 2.5 at diagnosis was a useful predictor of poor OS, regardless of the subsequent treatment (26). In HR+/HER2- eBC, high NLR (>2.25) after neoadjuvant chemotherapy was correlated with poorer disease free survival (DFS) and OS, especially in patients with non-pathologic complete response (pCR) (15). No consensus has been reached to define a cut-off or threshold value for each factor (NLR, lymphopenia, PLR or LMR). We first determined these cut-offs with ROC curves. In our study, NLR cut-off was similar to those found in the literature mostly ranging between 2 and 5. A meta-analysis conducted in BC reported a median NLR cut-off value of 2.5 in 10 out of 15 studies (13). Among different parameters studied, NLR was the only biomarker to show a difference on OS. The median PFS of 27 months in our study was similar to that expected and obtained in the registration trials of CdK4/6 inhibitors (5, 27, 28). To our knowledge, until now our study is the first to report significative prognostic impact of NLR on survival and a benefit in response rates to first line CdK4/6i and ET for HR+/HER2- mBC.

Cell death secondary to breast tumor cells expressing pro-apoptotic ligands and reduced thymic function have been suggested as possible mechanisms of peripheral lymphopenia observed in metastatic patients (29). Lymphopenia and NLR are two complementary prognostic factors. Lymphopenia is multifactorial and can be associated with patient characteristics (age, ECOG-PS) (30) or tumor burden and evolves with previous therapies. Increased systemic inflammation markers have been reported in lymphopenic patients, with an inverse increase in the percentage of peripheral neutrophils in response to the expression of pro-inflammatory cytokines such as IL-6 and IL-7, CD4+CD8+ double-positive (DP) thymocytes and an age-related decrease in thymic function or combinatorial T cell receptor diversity (30). The median age was similar between the two groups of pretreatments NLR, but they differed on ECOG-PS: patients in the high NLR group had a worse ECOG-PS. After adjustment on ECOG-PS, NLR was still significantly associated with poorer PFS.

In our study, we observed that more patients with high NLR received radiotherapy within the previous 90 days. This may be explained by the fact that radiotherapy induced lymphopenia can persist for several months (31). In multivariate analysis, we observed that the occurrence of radiotherapy was not associated with PFS. In our study, radiotherapy is not an independent poor prognostic factor and the prolonged lymphopenia may be multifactorial, partly secondary to cancer itself. Systemic treatments (corticosteroid therapy, chemotherapy) could also alter NLR and ALC. We did not observe significant difference regarding corticosteroid therapy between the two NLR groups at baseline. Only one patient in each NLR group received chemotherapy in the months before introduction CdK4/6i but none had presented a disease progression at the time of analysis.

Tumors are infiltrated by leucocytes and produce cytokines and chemokines. Lymphocytes, whether in peripheral blood or as tumor-infiltrating lymphocytes, play a major role in controlling disease progression. In a population of HR+/HER2 mBC patients already treated at least for one metastatic line, we previously showed that those with pretherapeutic ALC< 1.5 G/L had significantly shorter PFS time (6 vs. 10 months, p=0.004), shorter OS time (20 vs. 33 months, p=0.018) and more disease progression at first imaging evaluation (32). The difference on PFS and OS was demonstrated from the onset of lymphopenia. For this reason, we have selected a lymphocyte count of 1.5 G/L to. Although the results are not significant probably due to the lack of power, our study provided further evidence that lymphopenia is a negative prognostic factor for PFS and OS for patients receiving CdK4/6i.

It is necessary to thoroughly understand the impact of the immune system on tumor control. On the one hand, neutrophils, B lymphocytes and some CD4+ T cells may stimulate cancer growth. On the other hand, cytotoxic CD8+ T cells are crucial components of tumor-specific cellular adaptive immunity as T­helper (TH) 1, TH17, CD4+ T cells and Natural Killer cells are in the tumor microenvironment are. They inhibit tumor growth by producing interferon gamma, subsequently leading to angiostasis, cell cycle inhibition, apoptosis and tumor phagocytosis by macrophages (9). A retrospective study of 1,902 patients with eBC showed that a high total and peripheral CD8+ T cell count was associated with significantly longer breast cancer-specific survival (BCSS) (33). More specifically, in patients with ER-positive tumor, the total number of infiltrating CD8+ T cells was not significantly associated with patient outcome, whereas peripheral CD8+ count was associated with longer BCSS (33). Furthermore, Coffelt et al. demonstrated that elevated neutrophil counts induced by BC tumor cells suppressed CD8+ T cells and promoted metastasis through immunosuppression (34). CdK4/6i have been reported to increase tumor immunogenicity by overcoming two principal mechanisms of tumor immune evasion. They limit the proliferation of regulatory T cells leading to reduced immunosuppression and enhance antitumor immunity by increasing T cell activation, promoting T cell tumor infiltration, and expanding the functional capacity of tumor cells to present antigens (35, 36). This may explain that NLR could be a good biomarker to predict survival and response to CdK4/6i.

Other biomarkers evaluated had no significant impact on survival. PLR has been described as a reliable prognostic marker in many cancers including BC (37, 38). Concerning LMR in the BC neoadjuvant chemotherapy setting, a recent study confirmed this result in a multivariable analysis and showed that patients with low LMR had shorter DFS (16).

Immune status is emerging as an essential biomarker of the tumor biology and microenvironment with an impact on patient outcome. Other biomarkers, such as tumor infiltrating lymphocytes (TILS) and circulating tumor cells (CTC), are still being evaluated in clinical research as prognostic factors but are not easily obtained in routine clinical practice (7).

Despite adjustment on confounding factors, our study had some limitations. Due to the retrospective nature, we were unable to collect the values of some inflammation parameters (albumin, C-reactive protein and LDH). Also we did not have complete information on other discriminating factors of immune response, such as number of B cells, T cells or CD4/CD8 ratio, as these are not routinely performed. The sample size is limited and results must be interpreted with caution. Especially, PLR and LMR were not significant on the primary outcome possibly due to a lack of power, but also because the cut-off determined was not sufficiently discriminating. Moreover, as previously mentioned, there is no NLR threshold recognized in the literature in either breast cancer or any solid cancer, possibly due to its recent identification as a potential prognostic factor. Thus, our 2.53 cut-off NLR obtained by ROC curves requires internal and external validation in future studies.

Our study is the first one concerning the NLR prognostic factor for HR+ HER2- mBC population in first line metastasis only, and treated in this setting with cdk4/6 inhibitors and endocrinotherapy. Our population is therefore notably homogeneous, that increasing the power. Indeed, other studies were interested in the NLR prognostic factor, but their population was inhomogeneous as they included patients at the localized and metastatic stage (39), or metastatic patients only and under cdk4/6 inhibitors but all lines combined without information on previous treatments (40, 41). In this sense, it is an original study.

Nevertheless, in view of these first interesting results, it prompted us to design a prospective study (NCT05303129) in order to complete, confirm and improve these results more powerfully.

## Conclusion

Our study highlights NLR as new interesting biomarkers for mBC patients treated with CdK4/6i in the first-line setting. It can be used in routine clinical practice related to it availability, easy-to-use, reliable and inexpensive prognostic factor. These results may allow us to identify different prognostic groups. There are currently few prognostic factors in mBC. To date, none have been validated and are commonly used in first-line metastasis in patients receiving CDK4/6i. Our next project is to validate our results in a prospective study (NCT05303129).

## Data availability statement

The data analyzed in this study is subject to the following licenses/restrictions: The datasets generated during and/or analyzed during the current study are not publicly available due to the medically confidential nature of the data but are available from the corresponding author on reasonable request. Requests to access these datasets should be directed to PR. rottier@baclesse.unicancer.fr.

## Ethics statement

Written informed consent was obtained from the individual(s) for the publication of any potentially identifiable images or data included in this article. This is an observational study. The local Research Ethics Committee has confirmed that no ethical approval is required. In accordance with the regulations regarding research involving human subjects, the present study was registered with corresponding data protection. Patients’ non-opposition to the use of their data was sought after verification of their vital status.

## Author contributions

GE, AS and PR contributed to the study conception and design. Material preparation, data collection and analysis were performed by PR. JL conducted the statistics. The first draft of the manuscript was written by RP and SA, and all authors commented on previous versions of the manuscript. All authors contributed to the article and approved the submitted version.

## References

[1] The global cancer observatory (2019).

[2] Dawood S Broglio K Ensor J Hortobagyi GN Giordano SH. Survival differences among women with de novo stage IV and relapsed breast cancer. Ann Oncol (2010) 21(11):2169−74. doi: 10.1093/annonc/mdq220 20427349 PMC2962259

[3] Gennari A André F Barrios CH Cortés J de Azambuja E DeMichele A. ESMO clinical practice guideline for the diagnosis, staging and treatment of patients with metastatic breast cancer. Ann Oncol (2021) 32(12):1475–95. doi: 10.1016/j.annonc.2021.09.019 34678411

[4] Cardoso F Senkus E Costa A Papadopoulos E Aapro M André F. 4th ESO–ESMO international consensus guidelines for advanced breast cancer (ABC 4). Ann Oncol (2018) 29(8):1634−57. doi: 10.1093/annonc/mdy192 30032243 PMC7360146

[5] Finn RS Martin M Rugo HS Jones S Im SA Gelmon K. Palbociclib and letrozole in advanced breast cancer. N Engl J Med (2016) 375(20):1925−36. doi: 10.1056/NEJMoa1607303 27959613

[6] Cardoso F Kyriakides S Ohno S Penault-Llorca F Poortmans P Rubio IT. Early breast cancer: ESMO clinical practice guidelines for diagnosis, treatment and follow-up. Ann Oncol (2019) 30(8):1194−220. doi: 10.1016/j.annonc.2021.09.019 31161190

[7] Cuyún Carter G Mohanty M Stenger K Morato Guimaraes C Singuru S Basa P. Prognostic factors in hormone receptor-Positive/Human epidermal growth factor receptor 2-negative (HR+/HER2–) advanced breast cancer: A systematic literature review. Cancer Manag Res (2021) 13:6537−66. doi: 10.2147/CMAR.S300869 34447271 PMC8384149

[8] Coussens LM Werb Z. Inflammation and cancer. Nature (2002) 420(6917):860−7. doi: 10.1038/nature01322 12490959 PMC2803035

[9] Pagès F Galon J Dieu-Nosjean MC Tartour E Sautès-Fridman C Fridman WH. Immune infiltration in human tumors: A prognostic factor that should not be ignored. Oncogene (2010) 29(8):1093−102. doi: 10.1038/onc.2009.416 19946335

[10] Guthrie GJK Charles KA Roxburgh CSD Horgan PG McMillan DC Clarke SJ. The systemic inflammation-based neutrophil–lymphocyte ratio: Experience in patients with cancer. Crit Rev Oncol Hematol (2013) 88(1):218−30. doi: 10.1016/j.critrevonc.2013.03.010 23602134

[11] Clarke SJ Chua W Moore M Kao S Phan V Tan C. Use of inflammatory markers to guide cancer treatment. Clin Pharmacol Ther (2011) 90(3):475−8. doi: 10.1038/clpt.2011.122 21775983

[12] Templeton AJ McNamara MG Šeruga B Vera-Badillo FE Aneja P Ocaña A. Prognostic role of neutrophil-to-Lymphocyte ratio in solid tumors: A systematic review and meta-analysis. JNCI J Natl Cancer Inst (2014) 106(6): dju124. doi: 10.1093/jnci/dju124 24875653

[13] Ethier JL Desautels D Templeton A Shah PS Amir E. Prognostic role of neutrophil-to-lymphocyte ratio in breast cancer: A systematic review and meta-analysis. Breast Cancer Res (2017) 19(1):2. doi: 10.1186/s13058-016-0794-1 28057046 PMC5217326

[14] Koh CH Bhoo-Pathy N Ng KL Jabir RS Tan GH See MH. Utility of pre-treatment neutrophil–lymphocyte ratio and platelet–lymphocyte ratio as prognostic factors in breast cancer. Br J Cancer (2015) 113(1):150−8. doi: 10.1038/bjc.2015.183 26022929 PMC4647546

[15] Koh YW Lee HJ Ahn JH Lee JW Gong G. Prognostic significance of the ratio of absolute neutrophil to lymphocyte counts for breast cancer patients with ER/PR-positivity and HER2-negativity in neoadjuvant setting. Tumor Biol (2014) 35(10):9823−30. doi: 10.1007/s13277-014-2282-5 24986572

[16] Ma Y Zhang J Chen X. Lymphocyte-to-Monocyte ratio is associated with the poor prognosis of breast cancer patients receiving neoadjuvant chemotherapy. Cancer Manag Res (2021) 13:1571−80. doi: 10.2147/CMAR.S292048 33623436 PMC7896736

[17] Patel DA Xi J Luo J Hassan B Thomas S Ma CX. Neutrophil-to-lymphocyte ratio as a predictor of survival in patients with triple-negative breast cancer. Breast Cancer Res Treat (2019) 174(2):443−52. doi: 10.1007/s10549-018-05106-7 30604000

[18] Ivars Rubio A Yufera JC de la Morena P Fernández Sánchez A Navarro Manzano E García Garre E. Neutrophil-lymphocyte ratio in metastatic breast cancer is not an independent predictor of survival, but depends on other variables. Sci Rep (2019) 9(1):16979. doi: 10.1038/s41598-019-53606-3 31740715 PMC6861311

[19] Ni XJ Zhang XL Ou-Yang QW Qian GW Wang L Chen S. An elevated peripheral blood lymphocyte-to-Monocyte ratio predicts favorable response and prognosis in locally advanced breast cancer following neoadjuvant chemotherapy. PloS One (2014) 9(11):e111886. doi: 10.1371/journal.pone.0111886 25372468 PMC4221197

[20] Hayes DF Ethier S Lippman ME. New guidelines for reporting of tumor marker studies in breast cancer research and treatment: REMARK. Breast Cancer Res Treat (2006) 100(2):237−8. doi: 10.1007/s10549-006-9253-5 16773436

[21] Schwartz LH Seymour L Litière S Ford R Gwyther S Mandrekar S. RECIST 1.1 – standardisation and disease-specific adaptations: Perspectives from the RECIST working group. Eur J Cancer (2016) 62:138−45. doi: 10.1016/j.ejca.2016.03.082 27237360 PMC5737786

[22] Wei B Yao M Xing C Wang W Yao J Hong Y. The neutrophil lymphocyte ratio is associated with breast cancer prognosis: An updated systematic review and meta-analysis. OncoTargets Ther (2016) 9:5567−75. doi: 10.2147/OTT.S108419 PMC5021064 27660475

[23] Liu X Qu JK Zhang J Yan Y Zhao XX Wang JZ. Prognostic role of pretreatment neutrophil to lymphocyte ratio in breast cancer patients: A meta-analysis. Med (Baltimore) (2017) 96(45):e8101. doi: 10.1097/MD.0000000000008101 PMC5690700 29137007

[24] Noh H Eomm M Han A. Usefulness of pretreatment neutrophil to lymphocyte ratio in predicting disease-specific survival in breast cancer patients. J Breast Cancer. (2013) 16(1):55. doi: 10.4048/jbc.2013.16.1.55 23593082 PMC3625770

[25] Wariss BR de Souza Abrahão K de Aguiar SS Bergmann A Thuler LCS. Effectiveness of four inflammatory markers in predicting prognosis in 2374 women with breast cancer. Maturitas (2017) 101:51−6. doi: 10.1016/j.maturitas.2017.04.015 28539169

[26] de la Cruz-Ku G Chambergo-Michilot D Torres-Roman JS Rebaza P Pinto J Araujo J. Neutrophil-to-lymphocyte ratio predicts early mortality in females with metastatic triple-negative breast cancer. PloS One (2020) 15(12):e0243447. doi: 10.1371/journal.pone.0243447 33284847 PMC7721150

[27] Hortobagyi GN Stemmer SM Burris HA Yap YS Sonke GS Paluch-Shimon S. Updated results from MONALEESA-2, a phase III trial of first-line ribociclib plus letrozole versus placebo plus letrozole in hormone receptor-positive, HER2-negative advanced breast cancer. Ann Oncol (2018) 29(7):1541−7. doi: 10.1093/annonc/mdy155 29718092

[28] Johnston S Martin M Di Leo A Im SA Awada A Forrester T. MONARCH 3 final PFS: a randomized study of abemaciclib as initial therapy for advanced breast cancer. NPJ Breast Cancer (2019) 5(1):5. doi: 10.1038/s41523-018-0097-z 30675515 PMC6336880

[29] Manuel M Tredan O Bachelot T Clapisson G Courtier A Parmentier G. Lymphopenia combined with low TCR diversity (divpenia) predicts poor overall survival in metastatic breast cancer patients. OncoImmunology (2012) 1(4):432−40. doi: 10.4161/onci.19545 22754761 PMC3382902

[30] Ferrando-Martínez S Franco JM Hernandez A Ordoñez A Gutierrez E Abad A. Thymopoiesis in elderly human is associated with systemic inflammatory status. AGE (2009) 31(2):87−97. doi: 10.1007/s11357-008-9084-x 19507053 PMC2693727

[31] Cesaire M Le Mauff B Rambeau A Toutirais O Thariat J. Mécanismes de la lymphopénie radio-induite et implications thérapeutiques. Bull Cancer (Paris) (2020) 107(7−8):813−22. doi: 10.1016/j.bulcan.2020.04.009 32451070

[32] Emile G Penager S Levy C Johnson A Allouache D Lequesne J. Baseline lymphopenia as prognostic factor in patients with metastatic breast cancer treated with palbociclib. Oncol Lett (2021) 23(1):25. doi: 10.3892/ol.2021.13143 34868362 PMC8630820

[33] Mahmoud SMA Paish EC Powe DG Macmillan RD Grainge MJ Lee AHS. Tumor-infiltrating CD8 ^+^ lymphocytes predict clinical outcome in breast cancer. J Clin Oncol (2011) 29(15):1949−55. doi: 10.1200/JCO.2010.30.5037 21483002

[34] Coffelt SB Kersten K Doornebal CW Weiden J Vrijland K Hau CS. IL-17-producing γδ T cells and neutrophils conspire to promote breast cancer metastasis. Nature (2015) 522(7556):345−8. doi: 10.1038/nature14282 25822788 PMC4475637

[35] Goel S DeCristo MJ Watt AC BrinJones H Sceneay J Li BB. CDK4/6 inhibition triggers anti-tumour immunity. Nature (2017) 548(7668):471−5. doi: 10.1038/nature23465 28813415 PMC5570667

[36] Deng J Wang ES Jenkins RW Li S Dries R Yates K. CDK4/6 inhibition augments antitumor immunity by enhancing T-cell activation. Cancer Discovery (2018) 8(2):216−33. doi: 10.1158/2159-8290.CD-17-0915 29101163 PMC5809273

[37] Templeton AJ Ace O McNamara MG Al-Mubarak M Vera-Badillo FE Hermanns T. Prognostic role of platelet to lymphocyte ratio in solid tumors: A systematic review and meta-analysis. Cancer Epidemiol Biomarkers Prev (2014) 23(7):1204−12. doi: 10.1158/1055-9965.EPI-14-0146 24793958

[38] Hu Y Wang S Ding N Li N Huang J Xiao Z. Platelet/Lymphocyte ratio is superior to Neutrophil/Lymphocyte ratio as a predictor of chemotherapy response and disease-free survival in luminal b-like (HER2–) breast cancer. Clin Breast Cancer (2020) 20:e403−9. doi: 10.1016/j.clbc.2020.01.008 32201163

[39] Wira Wiguna IG Indrani Remitha NP Sadvika IGAS Wiranata S Putra IW Adiputra PAT. Pretreatment leukocyte count ratios as metastatic predictive factors in luminal type breast cancer. Asian Pac J Cancer Prev (2022) 23(5):1595−601. doi: 10.31557/APJCP.2022.23.5.1595 35633543 PMC9587872

[40] Shikanai A Horimoto Y Ishizuka Y Uomori T Nakai K Arakawa A. Clinicopathological features related to the efficacy of CDK4/6 inhibitor-based treatments in metastatic breast cancer. Breast Cancer Basic Clin Res (2022) 16:117822342110651. doi: 10.1177/11782234211065148 PMC8738870 35002243

[41] Yilmaz H Nigdelioglu B Aytac A Turan M Oktay E Yersal O. The prognostic importance of glucose-to-lymphocyte ratio and uric acid in metastatic breast cancer patients treated with cdk 4/6 inhibitors. Future Oncol (2022) 18(27):3043−53. doi: 10.2217/fon-2022-0464 36062468

