# Supplementary material for: Pretreatment neutrophil to lymphocyte ratio as prognostic factor in metastatic breast cancer treated with cyclin dependent kinase 4/6 inhibitors
Source: Front Oncol. 2023 Jan 19;12:1105587. doi: 10.3389/fonc.2022.1105587 (PMC9893782; doi:10.3389/fonc.2022.1105587)
Supplement: Supplementary file 1 [file Table_1.docx]

| Variables | Univariable | | Multivariable | | Selected model | |
| --- | --- | --- | --- | --- | --- | --- |
|  | OR [95% CI] | p | OR [95% CI] | p | OR [95% CI] | p |
| NLR < 2.53 | 0.50 [0.31-0.79] | 0.003 | 0.59 [0.33-1.08] | 0.089 | 0.57 [0.36-0.90] | 0.016 |
| Lymphopenia | 1.52 [0.97-2.39] | 0.071 | 1.12 [0.62-2.04] | 0.71 |  |  |
| Grade 3 or 4 toxicity | 0.73 [0.47-1.14] | 0.17 | 0.81 [0.48-1.36] | 0.42 |  |  |
| De novo metastatic disease | 0.94 [0.58-1.53] | 0.80 | 0.99 [0.54-1.80] | 0.96 |  |  |
| Bone metastase | 1.54 [0.85-2.81] | 0.16 | 1.14 [0.55-2.37] | 0.72 |  |  |
| SBR (ref = Grade 1) |  |  |  |  |  |  |
| Grade 2 | 0.83 [0.43-1.61] | 0.57 | 1.19 [0.56-2.54] | 0.65 |  |  |
| Grade 3 | 1.09 [0.53-2.24] | 0.82 | 1.57 [0.71-3.44] | 0.26 |  |  |
| PR positive | 0.75 [0.46-1.21] | 0.23 | 0.82 [0.48-1.40] | 0.47 |  |  |
| ECOG PS 1, 2 or 3 | 2.52 [1.52-4.16] | <0.001 | 2.36 [1.34-4.15] | 0.003 | 2.28 [1.37-3.79] | 0.001 |
| RT within 90 days before treatment | 1.49 [0.90-2.46] | 0.12 | 0.95 [0.50-1.79] | 0.87 |  |  |

Supplementary Table 1 :
